# Secure Publish-Subscribe Protocols for Heterogeneous Medical Wireless Body Area Networks

**DOI:** 10.3390/s141222619

**Published:** 2014-12-28

**Authors:** Pablo Picazo-Sanchez, Juan E. Tapiador, Pedro Peris-Lopez, Guillermo Suarez-Tangil

**Affiliations:** Department of Computer Science, Universidad Carlos III de Madrid, 28911 Leganes, Madrid, Spain; E-Mails: ppicazo@inf.uc3m.es (P.P.-S.); pperis@inf.uc3m.es (P.P.-L.); guillermo.suarez.tangil@uc3m.es (G.S.-T.)

**Keywords:** wireless body area networks (WBAN), attribute-based encryption, eHealth security and privacy, wearable sensors

## Abstract

Security and privacy issues in medical wireless body area networks (WBANs) constitute a major unsolved concern because of the challenges posed by the scarcity of resources in WBAN devices and the usability restrictions imposed by the healthcare domain. In this paper, we describe a WBAN architecture based on the well-known publish-subscribe paradigm. We present two protocols for publishing data and sending commands to a sensor that guarantee confidentiality and fine-grained access control. Both protocols are based on a recently proposed ciphertext policy attribute-based encryption (CP-ABE) scheme that is lightweight enough to be embedded into wearable sensors. We show how sensors can implement lattice-based access control (LBAC) policies using this scheme, which are highly appropriate for the eHealth domain. We report experimental results with a prototype implementation demonstrating the suitability of our proposed solution.

## 1. Introduction

The development of reasonably powerful wearable sensors and medical devices has stimulated research in wireless body area networks (WBANs) applied to healthcare scenarios. A prototypical scenario is that of a patient equipped with a number of wearable and implantable sensors that constantly measures various health-related parameters. Sensors are networked, meaning that they have communication capabilities and can interact with each other and with a central network controller that provides coordination, long-term storage, *etc*. The WBAN is often assumed to possess the ability to connect with external entities, for example, through an Internet connection. This would allow healthcare staff to monitor the patient remotely, continuously and in real time [1], even using automatically generated prognoses of the patient's health conditions with methodologies, such as the one proposed in [2]). Overall, the possibilities offered by WBAN technologies in the healthcare domain are potentially huge, ranging from the ubiquitous provisioning of healthcare services to enhanced emergency medical response systems and technologies to promote healthier living styles.

Wearable and implantable medical sensors and devices constitute an already established industry. For example, the market of implantable medical devices (IMDs) has been progressively growing year after year, and it is expected to be worth more than $43 billion in 2011 and more than $70 billion in 2018, according to research made by Transparency Market Research [3]. IMDs are usually given as small microchips located inside the human body to perform some medical-related function. The most common include pacemakers, defibrillators, cochlear implants, insulin pumps and neurostimulators. In their current generation (or in the near future), all of them share a common feature: wireless communication capabilities [4]. Moreover, IMDs have the ability to support and store telemetry data, facilitating the remote monitoring of the patient. IMDs can be part of a WBAN, operating both as sensors and as actuators and making decisions in real time.

In recent years, the proliferation of smartphones and other mobile “smart” devices with substantial computational and communication capabilities have reshaped the way WBANs may be implemented. Many works place a smartphone as the WBAN central node, using Bluetooth and Wi-Fi connections to group together all wearable sensors and devices. Apps running on the smartphone and other smart wearable devices provide an interface to access sensor data, which can be forwarded to healthcare staff using the smartphone Internet connection. Offloading computing and storage capabilities to the cloud has also been suggested to overcome the limitations of wearable devices [5,6].

Security and privacy issues have been described as two of the most challenging problems of IMDs and, more generally, WBANs [1,7–9]. As an example, it has been demonstrated that somebody equipped with a low-cost device can eavesdrop on the data communicated with a pacemaker and may even induce a cardiac arrest [10]. Health-related data have been the focus of several attacks almost since the adoption of computers in the healthcare domain. As a recent example, in 2010, personal data from more than 26 million veterans were stolen from the Department of Veterans Affairs' database in the USA by an employee who had access to the database [11]. The Ponemon Institute pointed out that Germany and the USA spent in 2013 more than $7.56 and $11 million, respectively, to protect personal health records from attacks. The most important security and privacy challenges in WBANs for healthcare scenarios include:

- Data confidentiality. Data generated in the WBAN is highly sensitive and must be encrypted both at storage and during transmission, so that users without the appropriate keys cannot access the data [12,13].
- Data integrity and authentication. It must be ensured that a message has been generated by a valid sensor and that it has not been tampered with by an adversary. Data integrity and authentication can be attained using standard cryptographic techniques in WBANs [1,12,13].
- Fine-grained access control. In this context, fine-grained refers to the granularity of the data access policy defined to specify and enforce different access privileges for different users. Trade-offs between access control and efficiency/usability must be considered, as a higher level of privacy discloses less information, but incurs more costs, while a lower privacy level leaks more details, but may be efficient [1,14,15].
- Software security. Code running in medical devices should be carefully designed and analyzed [12]. Software vulnerabilities in a WBAN sensor or actuator may have serious consequences for the patient's privacy and, in some cases, even lead to life-threatening situations.
- Limited capabilities. Most implantable and wearable devices are battery-operated and suffer from severe restrictions in their computing and communication capabilities. Thus, while many traditional embedded systems can rely on cryptographic measures without limitations, this must be carefully considered for implantable and wearable medical devices [1,7].
- Realistic threat and operation models. Currently, there are no clearly established models for the typical operation mode of a WBAN and the associated threat model(s). For example, it seems clear that a compromise of one WBAN node (e.g., if it is lost or stolen) should not put at risk other data or devices [1,12], but more comprehensive security models are needed. Similarly, it is unclear how to manage critical medical situations in which unauthorized users (e.g., paramedics, doctors belonging to a foreign hospital, *etc.*) can detect the presence of medical devices, get immediate access to them and even be able to switch them off or reconfigure them [7]. How to efficiently and securely deal with this is still an open problem.
- Availability. Sensory data and wearable medical services must be available at all times. More importantly, data and services should be able to dynamically adapt to contexts, such as time, location or certain events related to patients, and this data should be correct, even under Byzantine node failure [1,13].

### 1.1. Overview and Contributions

In this paper, we introduce a WBAN architecture based on the publish-subscribe messaging paradigm for wearable and implantable sensors and devices. The WBAN is thus viewed as a shared bus where a number of entities—sensors, apps residing in wearable smart devices, external users, *etc.*—produce data and subscribe to the data feed provided by other entities. We present two protocols for publishing data and sending commands to a sensor that guarantee confidentiality and fine-grained access control. Our protocols are based on a recently proposed ciphertext policy attribute-based encryption (CP-ABE) scheme that is lightweight enough to be embedded into wearable sensors [16].

Contrarily to other WBAN papers based on CP-ABE schemes, in our architecture sensors can encrypt data, but also decrypt messages generated by other devices. This allows for a flexible, scalable and highly versatile architecture, where services can be dynamically composed by subscribing to the data feeds published by wearable sensors. One major restriction of our chosen CP-ABE scheme is that only AND-based policies can be formed. Nonetheless, we show that this suffices to implement lattice-based access control (LBAC) [17] policies, which are highly appropriate for the eHealth domain.

The rest of this paper is organized as follows. In Section 2, we provide some background on ABE techniques and, in particular, on CP-ABE. Our proposed solution is described in Section 3 and evaluated in Section 4, both in terms of security and experimental efficiency. Section 5 provides an overview of related work in WBANs for healthcare applications. Finally, Section 6 concludes the paper and discusses our ongoing and future work in this area.

## 2. Preliminaries

For completeness and readability, we next provide a brief overview of the cryptographic primitives used in the protocols proposed in this paper.

### 2.1. Attribute-Based Encryption

Attribute-based encryption (ABE) was firstly presented by Sahai and Waters in [18] as a new way to provide authenticated users with encrypted access control. ABE is a type of public cryptography technique where messages are encrypted with both a private key and a ciphertext that correspond to the user's public attributes. Data can be decrypted by everyone whose attributes satisfy the policy set by the encryptor. Traditionally, the cost of these schemes in terms of computation, private key size and ciphertext size increases exponentially with the number of the attributes used. However, recent advances have demonstrated that even some lightweight devices, such as RFID labels, can implement ABE decryption [16]. Additionally, ABE cryptography is one of the most suitable cryptographic ways to provide access control while having low computation and storage overhead [1].

ABE schemes can be categorized into four different types:

- Key-policy ABE (KP-ABE) was proposed by Goyal *et al.* [19] in 2006 to achieve fine-grained access control in a more flexible manner than ABE schemes. KP-ABE introduces more complex access structures (policies) to encrypt messages: Boolean formula, including AND and OR operations. Additionally, each decryption key is based on a set of public attributes *S*. Finally, a user who wants to decrypt a message must match her attributes with the ciphertext. This is a disadvantage, because the owner cannot choose who is able to decrypt messages.
- Non-monotonic ABE was proposed by Ostrovsky *et al.* [20] in 2007. In this work, the authors extended the traditional ABE scheme by introducing a Boolean formula where AND, OR, NOT and threshold operations are available. The scheme has overhead problems, because of negative clauses, which make it infeasible to be developed in constrained devices.
- Ciphertext-policy ABE (CP-ABE) was proposed by Bethencourt *et al.* [21] in 2007. The authors presented an ABE scheme that corrects one of the disadvantages of KP-ABE, namely the ability of choosing who will be able to decrypt messages. To do so, the authors switch encryption and decryption algorithms, including the attribute set *S*, into the ciphertext and a policy into the key With this change, the ciphertext is encrypted with a tree access policy, and users who want to decrypt a message must match a set of attributes. The scheme's main disadvantage is a high computational cost in the decryption algorithm, particularly if *S* is large, since the more attributes the policy has, the higher the tree is.
- Hierarchical attribute-based encryption (HABE) was proposed by Wang *et al.* [22] in 2011 and uses policies in disjunctive normal form (DNF), where disjunctions are used to express the access control policy and conjunctions are used to manage all attributes. The scheme does not allow one to define fine-grained access control policies, but this can be achieved by combining both HIBE (Hierarchical Identity-Based Encryption) and CP-ABE. HABE is unsuitable to be implemented in real systems, because it is assumed that all attributes in one conjunctive clause may be managed by the same authority, which may cause the same attribute to be managed by multiple authorities.

In 2011, Waters developed a general method to construct a CP-ABE scheme using linear secret sharing techniques [23]. This is the most efficient scheme to date. Additionally, in order to solve the high computational cost that decryption involves, Green *et al.* [24] proposed to offload ABE decryption (KP-ABE and CP-ABE) to an external cloud server. To do so, the authors transform an ABE ciphertext satisfied by a particular set of user attributes into a constant-size ciphertext.

In our work, we rely on CP-ABE schemes for two main reasons: (i) it is the most suitable option when there are computational constraints [16,23]; and (ii) the party who encrypts the message chooses who can access the data [21].

### 2.2. CP-ABE Definitions

We next provide a brief background on CP-ABE schemes. We first introduce the notion of access structure, then describe bilinear maps and the variation of the Diffie-Hellman algorithm, known as augmented multi-sequence of exponents decisional Diffie-Hellman (aMSE-DDH), used in this work and, finally, discuss the security model of CP-ABE.

#### 2.2.1. Access Structure

We denote by 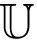 the attribute universe description and by 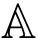 a collection of attributes {*A*_1_, *A*_2_, …, *A_n_*}, with *A_i_* ∈ {0, 1}. 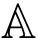 is an access structure over 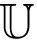 given by a collection of non-empty subsets of 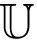, where the sets specified by 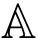 are called the authorized sets. Each time a user joins the system, a list of attributes is assigned to him, implicitly indicating what privileges he will have in the system.

#### 2.2.2. Bilinear Pairings

##### Definition 1

*Let p, r be two different primes, G an elliptic group, g a generator of G and e a bilinear map: e* : *G* × *G* → *G with the next properties:*

- *Bilinear: ∀_u_, υ* ∈ *G and a, b* ∈ *ℤ_p_; we have e*(*u^a^, υ^b^*) = *e*(*u,υ*)*^ab^*.
- *Non-degenerate: e*(*g,g*) ≠ 1.
- *Efficient: there exists an efficient algorithm to calculate e*(*u, υ*)∀*u, υ* ∈ *G*.
- *Symmetric: e is symmetric since e*(*g^a^, g^b^*) = *e*(*g, g*)*^ab^* = *e*(*g^b^, g^a^*).

#### 2.2.3. aMSE-DDH

The augmented multi-sequence of exponents decisional Diffie–Hellman (aMSE-DDH) problem is a slight modification of the multi-sequence of exponents decisional Diffie-Hellman problem considered in [25].

##### Definition 2

*Let x, y, z be three integers. As demonstrated in [26], for any probabilistic algorithm* 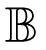 *making at most n queries using bilinear groups of prime order p, the advantage in solving the aMSE-DDH problem is*:

$$Ad\upsilon_{\text{B}}^{\left(x,y,z)-(aMSE-DDH\right)}(\lambda )=\frac{{\left(n+2s+2\right)}^{2}\cdot d}{2p}$$

where *s* = 4*y* + 3*x* + *z* + 3 and *d* = *max*{2(*x* + 2), 2(*y* + 2), 4(*y* − *z*) + 10}

### 2.3. CP-ABE Algorithms

A CP-ABE scheme implements four polynomial-time algorithms: Setup(), KeyGen(), Encrypt() and Decrypt(). Additionally, some CP-ABE schemes implement a fifth method, named Delegate(), which is used to give temporal access to a given user who is usually not allowed to access that information.

- Setup(λ, 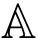). This method requires as input both a security parameter λ and the number of attributes defined in the system. It outputs two parameters: a public parameter *PK* and a master key *MK*.
- KeyGen(*MK*, *S*). This method requires as input both the master key *MK* and a set of attributes *S* that describe the key. It returns a private key *SK*.
- Encrypt (*PK, M*, 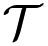). This method requires as input three values: the public parameters *PK*, the message *M* and the access structure 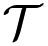. The algorithm encrypts *M* and outputs a ciphertext *C*_
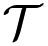
_, which will only decrypt if and only if the user's attributes satisfy the access structure. We assume that 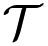 is implicitly included in *C*_
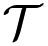
_.
- Decrypt(*PK*, *C*_
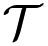
_, *SK*). This method requires as input three values: the public parameters *PK*, a ciphertext *C*_
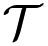
_ (with the access policy) and a private key *SK* for an attribute set. The method returns a decrypted message *M* only if the set of attributes satisfies the access structure embedded in *C*_
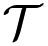
_; otherwise, it will return the error symbol ┴.
- Delegate(*SK*, *Ŝ*). This method requires as input a secret key *SK* (associated with a set of attributes *S*) and another set *Ŝ*, such that *Ŝ* ⊆ *S*. It outputs a private key $\hat{SK}$ for the set *Ŝ*.

### 2.4. Security Model

The chosen plain text attack (CPA) security model is based on the IND-sAtt-CPA game, which is a simulation where the adversary tries to attack an encrypted message without a decryption key, the attributes of which satisfy the message access policy. The game between an adversary and a challenger is described as follows.

#### Definition 3

*A CP-ABE scheme is said to be secure against an adaptive chosen plain text attack (CPA) if any polynomial-time adversary has only a negligible advantage in the IND-sAtt-CPA game, where the advantage is defined to be Adυ* = |*Pr*[*b*′ = *b*] − 1|.

- Setup: The challenger starts the algorithm and runs the Setup() method to generate a key pair (*PK, SK*) with a security parameter λ, and sends *PK* to the adversary.
- Phase 1: For each attribute *A_i_* ∈ 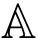, the adversary gets its secret key *SK_i_* by making requests to the KeyGen() method. The adversary cannot ask for a *A_i_* ∉ 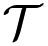, where 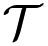 is his access structure.
- Challenge: The adversary creates two messages *M*_0_ and *M*_1_ with *len*(*M*_0_) = *len*(*M*_1_) and an access structure *
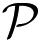
*. Because this structure cannot be satisfied by any *SK_i_*, the challenger picks a random *r* ∈ {0, 1} and returns the result (*C*) of the method Encrypt(*PK*, *M_r_*, 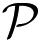).
- Query: The adversary can continue querying the KeyGen() method with the same restriction as in Phase 1.
- The adversary finally gets a guess for *r*: r* ∈ {0, 1} and wins the game if r* = *r*.

The advantage of an adversary is defined by $Ad\upsilon =Pr[r*=r]-\frac{1}{2}$.

#### Definition 4

*The CP-ABE scheme is fully secure against chosen ciphertext attack (CCA-secure) if all polynomial time adversaries have only a negligible advantage for* λ *in this game, i.e.*, $Pr[\text{CP}-\text{ABE}(\lambda ,\text{U})=1]\leq \frac{1}{2}+\text{negl}(\lambda )$

It is worth noting that a CP-ABE scheme has all of the properties defined in [27] and can be easily adapted to be secure against selective security by adding an initialization phase where the attacker must declare 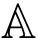 before seeing *PK*. Additionally, it is secure against chosen plain text attack (CPA-secure), because calls to Decrypt() are not allowed in Phases 1 and 2 above.

## 3. Our Solution

We next describe our proposed solution. We first provide an architectural overview and discuss the system model. We next describe the three procedures supported in our scheme: setup, publish and command protocols.

### 3.1. Architecture and System Model

Our solution considers a BAN composed of heterogeneous devices in terms of computational and communication capabilities. We assume that many of them are equipped with sensors that provide a number of physical and physiological parameters of the bearer, such as the electrocardiogram (ECG), galvanic skin response (GSR), temperature, heart rate, position, *etc*. Some devices could be “smart”, meaning that they can execute third-party apps (e.g., a smartphone or a smartwatch), while others could just be wearable or implantable sensors with limited functionality.

At a high level, our BAN uses a publish-subscribe architecture [28]. This is a well-known message-oriented system in which parties (*i.e.*, BAN nodes) can play two different roles: nodes that create new events are called publishers, and nodes who consume events are called subscribers. Note that in our model, “node” is an application-layer entity and should not be viewed as a physical device. For example, a powerful device with various sensors may support various apps running on it, each one publishing a different sensed signal. Similarly, a device may host several subscribers and no provider (e.g., a portable monitor running various apps that provide the bearer with information about his state).

This architecture presents several advantages. For instance, it makes it possible for one sensor to subscribe to the data feed published by another sensor and produce an output that depends on it. This allows for more complex functions to be embedded into wearable devices. For example, a heart rate sensor could subscribe to a location sensor (*i.e.*, GPS) and provide data correlated to the bearer's speed. Furthermore, it makes it possible for a sensor to have access to a signal whenever there is another sensor that publishes it in the BAN. Finally, it provides good scalability and flexibility, allowing dynamic topologies among sensor services and, therefore, very powerful applications based on fusing and processing different signals.

**Figure 1.**
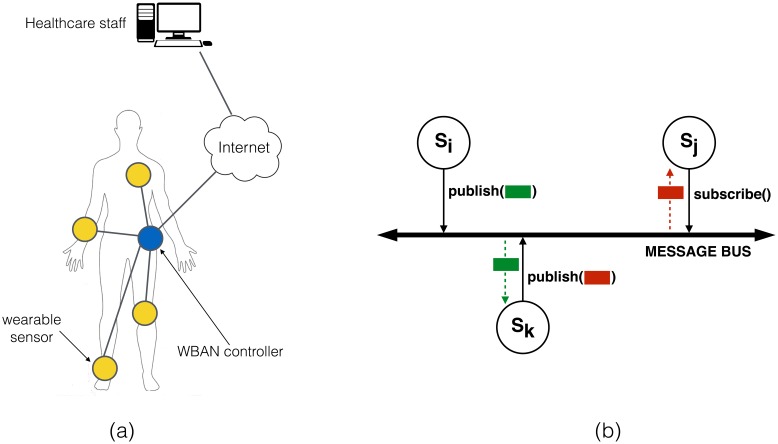
WBAN architecture: (**a**) physically, as a network of wearable devices; (**b**) logically, as a publish-subscribe messaging system.

In summary, our WBAN architecture can be seen at three different levels (see Figure 1):

- At the physical and network layer, devices will typically organize in a star topology where each node directly communicates with a network hub. This is the traditional approach in most WBANs, with the network hub being a dedicated network controller or, more recently, a smartphone. For reasons that will be clearer later, it is important that such a WBAN controller has sufficient computational resources. The hub will also act as the gateway for accessing external services (*i.e.*, the Internet or other devices in proximity of the WBAN) and, in many cases, will also provide storage capabilities to other sensors. This, however, can be delegated to another device.
- At the middleware layer, we refer to “entities” rather than to physical sensors or devices. The WBAN is seen as a collection of such entities connected by a (logical) shared bus. The bus is managed by the network hub or any other distinguished element, which provides each entity with a logical view of the architecture through the four classic methods in these architectures [29]: publish(), subscribe(), unsubscribe() and notify(). Each entity (e.g., a sensor) generates data asynchronously according to its configuration and capabilities. Such data is sent to the bus controller through the publish() method, which stores and forwards it to registered subscribers. The particular way in which such transmissions take place depends heavily on the underlying network technologies. For example, if a smartphone plays the role of WBAN controller, one sensor may connect to it using Bluetooth, while others may use Wi-Fi.
- Finally, at the application layer, we see the WBAN as a collection of sensing services running over different physical nodes. Each service provides a data feed to interested subscribers. Subscribers can be other services running in the WBAN or external entities, such as, for example, a doctor or a nurse in the case of a medical application. In such a case, access to the WBAN will typically take place through the BAN controller, for example using the Internet as communication channel. External entities access services just as a WBAN entity would do it, *i.e.*, using the publish(), subscribe(), unsubscribe() and notify() methods.

#### 3.1.1. Securing Information Flows with Ciphertext Policies

The central aspect of our proposed solution is a fine-grained distributed access control scheme using a lightweight CP-ABE scheme [16]. This is a key security service in healthcare applications of WBANs, since unauthorized access to the data provided by medical sensors may compromise the user's privacy. In our scheme, each sensor is configured with a policy service that determines what attributes an entity must possess in order to access the data. Such a policy may be fixed (e.g., you need to be a doctor or a nurse to access data published by an ECG sensor) or may depend on the context (e.g., location, state of the patient, readings of other sensors, *etc*.).

The common approach in WBANs to grant access rights to patient-related data is to follow a role-based access control (RBAC) model [1]. In a healthcare setting, an RBAC approach classifies users according to their professional roles (e.g., doctors, nurses, admin staff, *etc.*) and defines policies based on those roles and, perhaps, on external conditions (context), too. CP-ABE supports policies with a tree-like structure, which are adequate to model expressive authorization sentences using roles and context parameters as attributes. Thus, whenever a WBAN sensor generates some data, it builds the ciphertext according to the appropriate access control policy for this particular piece of data.

One major restriction of using the scheme proposed in [16] is that it only supports AND policies. This restricts the types of policies supported in our proposal, although the possibility of having decryption services on-board allows for more complex decision-making, since some sensors can decrypt what others publish. Rather than using roles, our current policies are based on lattice-based access control (LBAC) [17]. LBAC is not significantly less expressive than RBAC and fits well with the idea of using only AND connectives in the policies. In LBAC, access control policies define a partial order and can be visualized as the Hasse diagram associated with the associated poset. A classical application of such policies is in multilevel security (MLS) systems, where data is labeled according to its sensitivity level using a number of classification levels (e.g., public, confidential, secret, top-secret). Moreover, in order to comply with the need-to-know principle, access to information should only be granted if it is necessary for the requester. This gives rise to the use of compartments. In a healthcare scenario, such compartments could correspond to departments or healthcare services.

**Figure 2.**
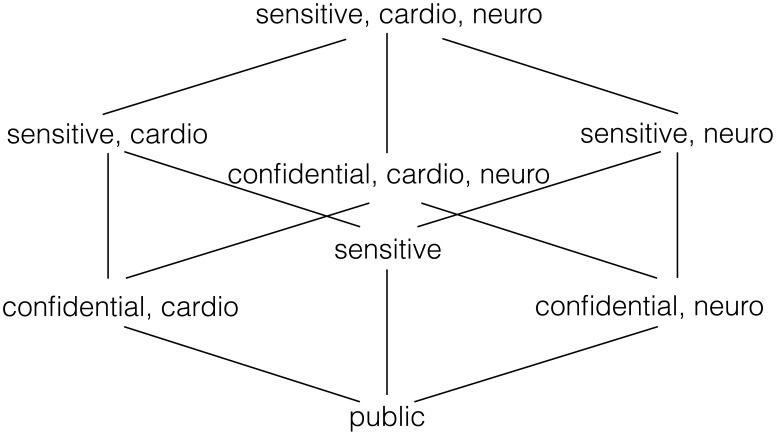
Hasse diagram for an example lattice-based access control (LBAC) policy using three security levels and two compartments.

We will use the following toy example to illustrate the type of LBAC policies supported in our system. Assume a WBAN composed of medical and sport sensors. Medical sensors can be grouped as cardiology-related and neurology-related. The information generated by the sensors belongs to three categories: public (e.g., the heart rate), confidential (e.g., the electrocardiogram (ECG) or the electroencephalography (EEG)) and sensitive. The combination of these levels and compartments give rise to the Hasse diagram shown in Figure 2. This could be implemented using a set of five attributes:

𝔸={public,confidential,sensitive,neuro,cardio}

so that each entity (sensors, apps and users) are provided with a key associated with a subset of 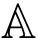. Note that if a user is given an attribute of Level *l*, he must be also given all attributes corresponding to the levels below.

Additional attributes can be created for specific privileges. For example, the ability to reconfigure a sensor can be explicitly modeled as a separate attribute.

### 3.2. Setup

Each entity (sensor, device, app, *etc.*) belonging to the WBAN needs to be initialized with the appropriate keys by a key generation center (KGC). The KGC is operated by the healthcare provider and produces the public parameters *PK* and a master key *MK* using the Setup(λ, 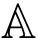) method with policy attributes 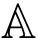. Each entity that wants to join the WBAN must be provided with *PK* and a secret key *SK* generated by the KGC using the KeyGen(*MK*, *S*) method, where *S* is the set of attributes (and, therefore, the access privileges) chosen for the entity.

Once initialized with the appropriate cryptographic material, the entity registers with the WBAN controller and retrieves the list of available sensors (publishers). After this, it can publish it owns contents and subscribe to other sensors' data feeds using the API provided by the messaging middleware.

### 3.3. Publish Protocol

**Figure 3.**
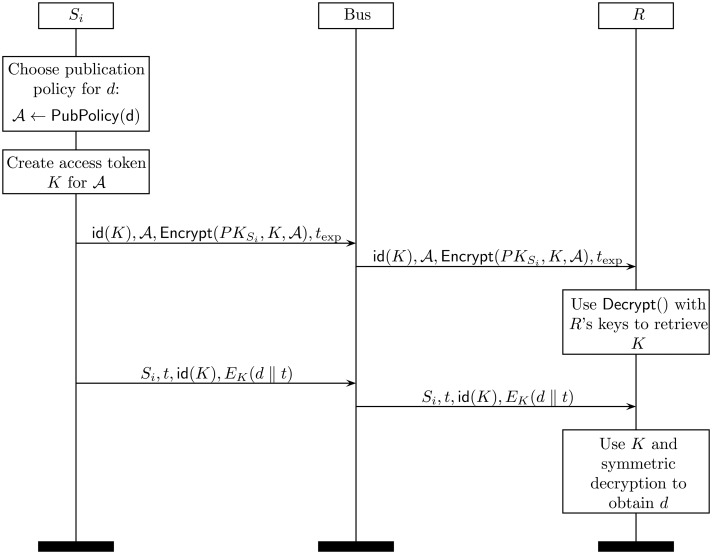
Publish protocol. Note that the access token is sent only once for every access structure.

When a sensor *S_i_* wants to publish data in the data bus, it follows the following procedure:

(1) Let *d* be the piece of data to be published. The sensor *S_i_* must determine under what access policy *d* will be published. We assume the existence of a policy service stored within the sensor that returns the access structure 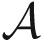 required for this particular piece of data:
  A←PubPolicy(d)
  Note that PubPolicy() may be as simple as a fixed access policy stored within the sensor, but also arbitrarily complex. For example, a powerful sensor may determine the access structure for a particular piece of data as a function of the location (e.g., whether at home, in the street, at the hospital, *etc.*), the time of the day, or even the physical state of the bearer. Thus, *S_i_* may need access to external sources of information, including other sensor in the BAN, to determine the context where the publication of *d* takes place.
(2) *S_i_* keeps a list of recently used access structures 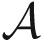 and the associated access token. An access token is just a symmetric key that will be required to actually get access to *d*. The list contains the following four elements:
  $$\left[\text{id}(K),A,\text{Encrypt}(PK_{S_{i}},K,A),t_{\exp}\right]$$
  where:
  - id(*K*) is the identifier of the access token (symmetric key) *K*.

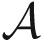
 is the data structure.
  - Encrypt(*PK_Si_*,*K*, 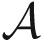) is the CP-ABE encryption of the symmetric key *K* using 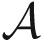.
  - *t*_exp_ is an expiration date after which this access token is no longer valid.
  After determining the access structure 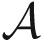 for this particular *d*, *S_i_* checks whether a unexpired access token is already available. If so, it retrieves it and uses that *K* in Step 3; otherwise, it creates a new one associated with 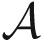 by randomly choosing a symmetric key *K*. The new access token is sent to the bus, so that it becomes available to already subscribed consumers:
  $$S_{i}\to \text{Bus}:[\text{id}(K),A,\text{Encrypt}(PK_{S_{i}},K,A),t_{\exp}]$$
(3) *S_i_* sends the following message to the bus:
  $$S_{i}\to \text{Bus}:[S_{i},t,\text{id}(K),E_{K}(d)\|t]$$
  where:
  - *S_i_* is the sensor's identity.
  - *t* is a timestamp.
  - id (*K*) is the identifier of the access token *K*.
  - *E_K_*(*d* ‖ *t*) is the symmetric encryption of *d* concatenated with *t* using key *K*.
(4) When a data consumer *R* who is subscribed to *S_i_*'*s* messages receives a new post, it checks id (*K*) and determines whether the corresponding access token is available or not. If this is the first message received with this access structure (e.g., because *R* has just subscribed to *S_i_*'*s* messages or because *S_i_* has changed the access policy for this piece of data), *R* must retrieve from *S_i_* the corresponding access token. This is done using the command protocol described in the next section. Once retrieved, it executes:
  $$\text{Decrypt}(PK_{R},\text{Encrypt}(PK_{S_{i}},K,A),SK_{R})$$
  to obtain *K* (if *R* has sufficient privileges) and, subsequently, the symmetric decryption *D_K_*(*E_K_*(*d*)) to retrieve *d* and check *t*.
  The entire protocol is illustrated in Figure 3.

### 3.4. Command Protocol

The command protocol implements the “get” and “set” functionalities common in many distributed services. It is used whenever a requester, either a device within the BAN or an external entity, commands a sensor *S_i_* to carry out an action. Such an action may be:

- A get() command in order to retrieve particular piece of data from the sensor. This could be, for example, the access token required to decrypt *S_i_*'s messages. Get operations are also useful to retrieve historical or statistical data stored in the sensor, as well as its configuration in a broad sense.
- A set() command, which is used to modify some configuration aspect of the sensor, including its sensing parameters, network configuration, security policies, *etc*.

The execution is essentially identical in both cases and consists of the following steps:

(1) The requester *R* selects an appropriate access structure 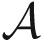 and a symmetric key *K* and sends to the target sensor *S_i_* the message:
  $$R\to S_{i}:[\text{Encrypt}({PK}_{R},K_{c},A),E_{K_{c}}(c\|t\|R\|S_{i})]$$
  where *c* is the get() or set() command with all of the associated parameters and *t* is a timestamp.
(2) Upon receiving the previous message, *S_i_* decrypts the first part
  $$\text{Decrypt}(PK_{S_{i}},\text{Encrypt}(PK_{R},K_{c},A),SK_{S_{i}})$$
  and obtains *K_c_*, which is used to decrypt the second part and get access to *c*. At this point, and after checking that *t* and the two identities are correct, *S_i_* checks whether *R* has sufficient privileges to require the execution of *c*. We assume the existence of a command policy service stored in the sensor that returns the privileges (*i.e.*, access structure) 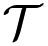 required to request the execution of *c*:
  T=CmdPolicy(c)
  Now, *S_i_* challenges *R* by sending the message:
  $$S_{i}\to R:[\text{Encrypt}({PK}_{S_{i}},(N\|t\|R\|S_{i}),T)]$$
  where *N* is a nonce.
(3) *R* decrypts the previous message, increases *N* and returns:
  $$R\to S_{i}:[\text{Encrypt}({PK}_{R},(N+1\|t\|R\|S_{i}),T)]$$
(4) *S_i_* decrypts the received message and checks that *N* is correct. If so, it executes *c* and sends back to *R* the response *r*(*c*) using the same access structure 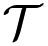:
  $$S_{i}\to R:[\text{Encrypt}(PK,(K_{r}\|t),T),E_{K_{r}}(r(c))]$$
  In the case of a get() command, *r*(*c*) contains the information requested by *R*. In the case of a set() command, *r*(*c*) may be a report about the execution or just an OK/error message.

Note that this protocol implicitly assumes that *R* and *S_i_* can communicate directly, hence the *R* → *S_i_* and *S_i_* → *R* notation. In practice, the WBAN controller will forward the message to the receiver using the appropriate signaling.

**Figure 4.**
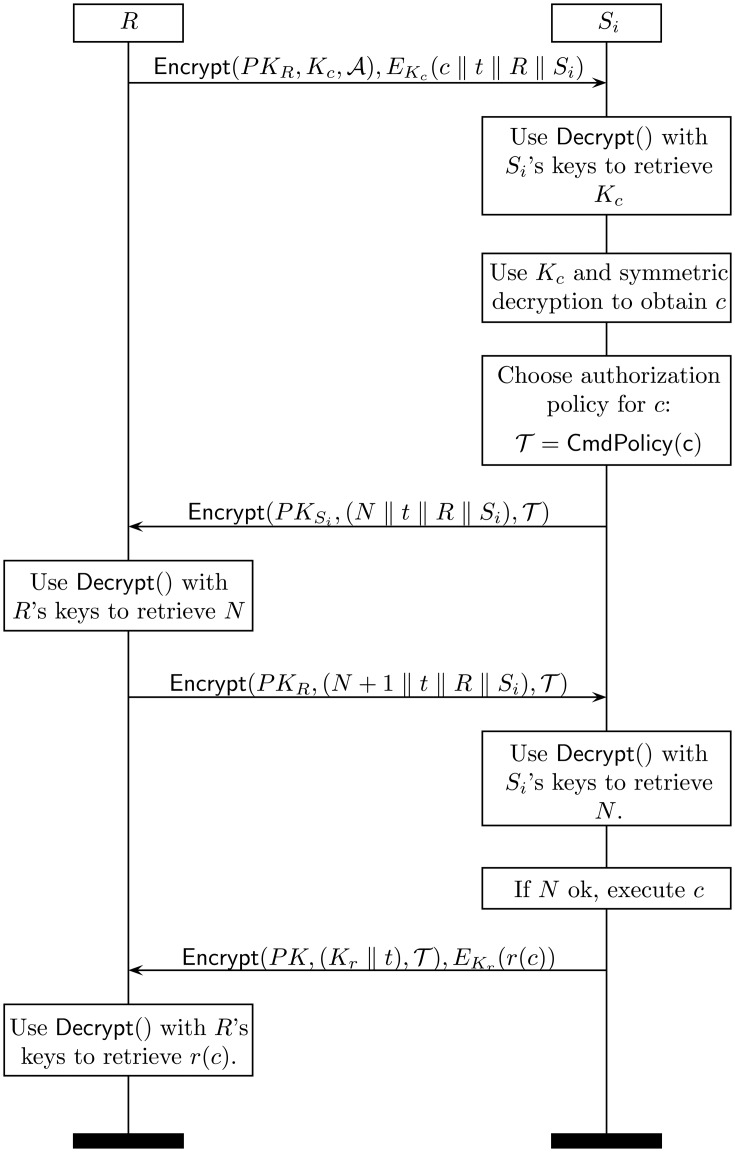
Command protocol.

The command protocol is illustrated in Figure 4.

## 4. Evaluation

In this section, we discuss the main security properties of the protocols introduced above and report experimental results about their efficiency obtained with a prototype implementation.

### 4.1. Security Analysis

#### 4.1.1. Data Confidentiality and Access Control

Confidentiality refers to the protection of sensitive information from being disclosed to unauthorized users. In our solution, we use a hybrid scheme, like the one used in PGP, with the aim of guaranteeing confidentiality while offering high efficiency. In the publish protocol presented above, session keys are protected through CP-ABE, and then, messages are symmetrically encrypted. Therefore, the security guarantees offered by CP-ABE and the strength of symmetric ciphers, like AES or 3-DES, allow us to claim that our solution does not put at risk confidentiality.

In our solution, we offer a fine-grained access control through LBAC polices. Although LBAC is less expressive than RBAC, in Section 3.1.1, we have shown how, using only AND connectives, we are able to define a broad set of policies. In particular, we propose the combined use of security levels and compartments, which helps us to provide wider expressibility despite being limited by using only one operator. On the other hand, the use of compartments suits the healthcare environment well.

#### 4.1.2. Resistance to Collusion Attacks

The use of CP-ABE guarantees resistance against collusion attacks in the following sense: if none of two data subscribers have sufficient privileges to successfully decrypt a ciphertext, but the union of their attributes do, it is impossible for them to somehow combine their secret keys to obtain one that can be used to decrypt the ciphertext. The impossibility of doing this is related to the use of different random numbers within each key.

#### 4.1.3. Authentication

We have not included authentication tokens, neither in the publish protocol, nor in in the command protocol. Authentication takes place at the middleware layer, so it is the bus controller that is in charge of verifying that a publisher is authentic before accepting a publication. This can be done in a standard way and is not the focus of this paper.

#### 4.1.4. Privacy within the WBAN

Untraceable communications are not one of our design goals in this paper. Consequently, it is possible for any entity with access to the bus to determine the identity of a sensor, when it publishes something, and even who is subscribed to what service. Avoiding this may be certainly interesting in many scenarios. However, anonymization measures are known to be quite expensive in traditional wireless sensor networks, so lightweight techniques suitable for a WBAN scenario would be welcome.

### 4.2. Performance

We next analyze and evaluate the performance between traditional management and our proposed protocol in terms of functionality, computation, communication and storage overhead.

Comparing both traditional and ABE public key cryptography, one of the major differences they present is related to key distribution. In traditional algorithms, the computational overhead is proportional to the users the system has, *i.e., O*(*n*), whereas in CP-ABE schemes, the computational overhead usually tends to be *O*(1). Moreover, there is another main difference between traditional and CP-ABE cryptography in terms of data access: the party who decrypts private data. In a traditional system, this operation is made by a trusted party (e.g., the bus controller in our case) before granting access to the final entity and then encrypting the ciphertext with the user's *PK*. This operation (encryption + decryption) increases the system's overhead, whereas in CP-ABE, such a trusted party only stores and forwards data. Decryption will only be made if the user's public attributes match with the access tree included in the ciphertext.

**Figure 5.**
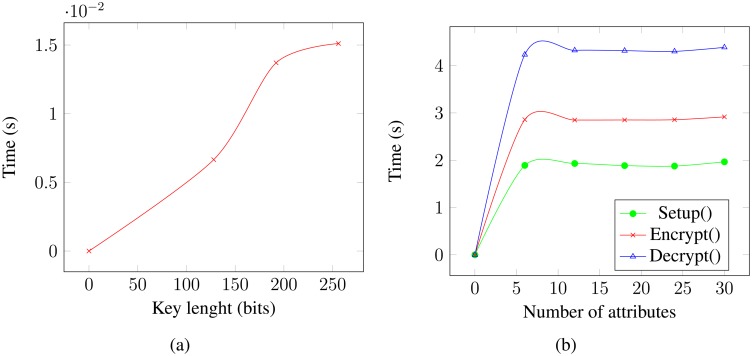
Execution time: (**a**) AES; (**b**) ciphertext policy attribute-based encryption (CP-ABE).

We have developed a prototype of our proposed solution for Android-based devices and run it on a Google Nexus 4 smartphone with a Qualcomm Snapdragon S4 Pro APQ8064 processor and 2 GB of RAM. To do this, we built an app that uses a Java implementation of both symmetric encryption/decryption and CP-ABE primitives, as in our publish and command protocols. Android v.4.4.4 was used in our tests. In a first round of experiments, we measured the time required by both symmetric and CP-ABE primitives. Figure 5 shows the time required by AES and CP-ABE to encrypt/decrypt 1 MB. The figures were obtained by averaging the result over 10 executions and show that encryption incurs little overhead. In particular, CP-ABE times are quite reasonable considering that in our solution, sensors only need to CP-ABE encrypt or decrypt when a new access token is required, which is a relatively infrequent event. Furthermore, access tokens consist basically of an AES key plus some metadata, which amounts to less than 1024 KB. Thus, CP-ABE encryption and decryption of an access token take roughly 3 to 4 ms.

### 4.3. Power Consumption

**Figure 6.**
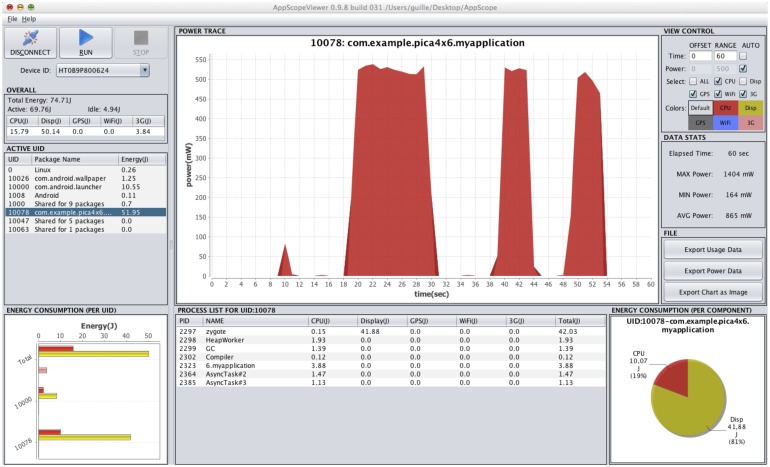
Power consumption trace of the CP-ABE Setup(), KeyGen(), Encrypt() and Decrypt() methods in an Android app.

In battery-powered sensors, power consumption is a major limitation, and security measures should not be very demanding in this regard. We have also measured the power consumption incurred by our solution when used in an Android platform. The experiments have been conducted by applying a battery of tests involving key generation, encryption and decryption operations. Our device was previously instrumented with AppScope [30], an energy metering framework based on monitoring kernel activity for Android. AppScope collects usage information from the monitored device and estimates the consumption of each running application using an energy model given by DevScope [31]. AppScope provides the amount of energy consumed by an app in the form of several time series, each one associated with a component of the device (CPU, Wi-Fi, cellular, touchscreen, *etc*.). We restrict our measures to CPU for computations, as our tests do not have a graphical user interface, do not require user interaction and, therefore, do not use any other component (see Figure 6).

Table 1 shows the results in terms of Joules per byte consumed by symmetric and CP-ABE encryption/decryption. As before, the figures are averages obtained over 10 executions. A noteworthy result is that the CP-ABE operations are around 1000-times more costly than their symmetric counterpart. This is reasonable and motivates designs like ours in which CP-ABE is only used to encrypt a symmetric key. In order to contextualize the energy implications of the previous figures, we have measured the power consumed by some popular apps during 10 min: watching multimedia content on YouTube, playing a game (MX Moto) and online social networking through Facebook (see Table 2). The amount of energy consumed ranges between approximately 550 J and 645 J, most of it being related to the graphical user interface.

## 5. Related Work

WBANs can be grouped into two different categories depending on whether they use an external device [5,32–34] or not [6,11,35–38]. The main disadvantage of using an external device is that the patient needs to wear it at all times. This increases the chances of it being stolen or lost, which could result in a compromise of all personal data stored on it. Thus, many research works have focused on schemes that do not rely on any external device. This architectures present two main challenges: how data is encrypted and how users can access data. The interested reader can find more information about WBANs in [39].

Bourbakis *et al.* have recently proposed in [40] a mobile health platform for secure information exchange in wearable health monitoring systems. The scheme incorporates various biometric authentication systems that are used to grant access to encrypted health data. Thus, the system incorporates authentication, authorization, confidentiality and integrity services. Contrary to our approach, the system in [40] is based on symmetric cryptographic primitives.

Barua *et al.* [35] proposed a scheme to control access to a patient's health information using different privacy levels. To do so, the authors use ABE in a rather standard way: privileges are mapped into roles and roles into ABE access structures. Additionally, cloud-based storage is used to reduce the cost and to allow data to be online anytime and anywhere. However, data is sent to the hospital server before storing a copy in the cloud. The hospital server becomes a bottleneck in this scheme, and no data is sent to the cloud if the server is down.

A similar protocol was presented by Akinyele *et al.* in [34]. The protocol uses ABE to generate self-protecting EHRs, which can either be stored on cloud servers or on cellphones, so that they could be accessed when the health provider is offline. Their solution is based on how personal health records are managed by the patients themselves using their mobile devices. The schemes involve a large number of messages exchanged between users and healthcare systems, and the existence of a single trusted authority that can decrypt all EHR is required. This creates a single point of failure, as the entire system would suffer a major privacy breach if this party is compromised.

In [5], the authors describe a prototype of a cloud mobile health monitoring system based on a WBAN and a smartphone. A neural network located as a cloud service is used to determine whether the patient is in danger. The scheme does not take into account the patient's privacy at any point, neither in the WBAN nor in the cloud, which makes it at least questionable regarding its applicability in real-world scenarios.

Yi *et al.* proposed in [38] a new protocol in which each sensor stores three different keys that are used to authenticate against three different data servers. If a third party wants access to the patient's data, it needs to obtain authorization from those three data servers.

Another work that uses a cloud server to reduce the decryption computation involved in IBE is the cloud-assisted mHealth monitoring system (CAM) [6]. This scheme consists of four main components: the cloud server, a company that provides the mHealth monitoring service, patients and a trust authority. As pointed out in [41], this work does not take into account the energy constraints of sensors and the real-time requirements of this kind of application.

Many recent works have focused on the problem of controlling access to specific data and assigning privileges to authorized users [11,14,36,37]. In [36], a WBAN is proposed to collect a large amount of data generated by medical sensor networks. The system makes use of a scalable cloud-based infrastructure to store and access the generated data in a secure way. In this work, the authors use CP-ABE and symmetric encryption to achieve fine-grained access with low computation overhead. A similar concept is proposed in [42], although in this work, the authors share devices instead of data, like in [36].

Another work based on CP-ASBE (Ciphertext Policy Attribute Set Based Encryption, which is an improved form of CP-ABE by introducing a recursive set-based structure on attributes associated with user keys) was presented in [37]. In this work, the authors proposed a scheme called CRYPE (Cryptographically enforced and Privacy enhanced) in order to guarantee the security and privacy of patients when somebody accesses data that have been previously stored in the cloud. Additionally, IBE is used for secure end-to-end communications. It is claimed that this protocol provides confidentiality, role-based access control with user revocation, scalability, flexibility and prevention of active attacks, such as DoS, and chosen ciphertext and plain text attacks.

Li *et al.* proposed an attribute revocation method for multi-authority ABE systems in [11] to reduce the overhead of key management. This means that the system is split into multiple security domains, each of which manages a subset of users. However, this scheme has two main issues: (i) it is only suitable for KP-ABE systems [43]; and (ii) it is a must that each patient generates and distributes her own security keys to the authorized users [36].

A work similar to ours is [44], which focuses on securing the communications between BAN sensors and external users using CP-ABE. Contrary to our publish-subscribe architecture, the work in [44] takes a data-centric approach in which a data sink receives data from all sensors. Furthermore, sensors can only encrypt and, therefore, cannot access data produced by another sensor.

The proliferation of networked WBAN medical devices has stimulated research on efficient architectures for cryptographic services. For example, the work in [45] proposes a system architecture for implantable devices where security and medical functionalities are decoupled by running them on two separate cores. The CP-ABE cryptosystem used in our work [16] constitutes another example of a lightweight scheme designed on-purpose to be embedded in mobile and wearable devices. Other works in this line include the SCAN secure processor [46], which supports biometric authentication and various symmetric encryption primitives.

## 6. Conclusions and Future Work

In this paper, we have introduced a publish-subscribe architecture for WBAN with particular emphasis on medical applications. In this domain, medical sensors producing highly sensitive information will likely coexist with devices intended for other purposes, such as sport or entertainment apps. We leverage the versatility offered by CP-ABE primitives to propose protocols that allow sensors to subscribe to the data feeds published by other sensors. The privileges required to access each particular datum are set by the sensor's policy, which can vary them depending on the context. Apps and external users (e.g., healthcare staff) can get access to such data feeds and also reconfigure or request specific data from the sensors, provided that they have sufficient privileges to do so. Our implementation of the underlying protocols make use of a recently proposed lightweight CP-ABE scheme. As a consequence of this, the entities use a constant size decryption key, which is independent of the used attributes. On the other hand, our scheme offers a fine-grained access control through LBAC policies that are limited to using AND operations only. Finally, it is worth mentioning that the proposed publish and command protocols facilitate modeling the principal interactions in a WBAN composed of a variable number of devices.

Our experimental results confirm that the scheme is suitable for most current sensors, including ARM-based platforms. We are currently building a full prototype using an Android smartphone as a WBAN controller and a publish-subscribe middleware based on Java Message Service (JMS). WBAN devices include various health sensors based on the Arduino platform (in particular, the e-Health Sensor Platform v2.0 by Cooking Hacks), including heart-rate monitors, galvanic skin response sensors, position sensors, ECGs, body temperature, *etc.* using various communication technologies (Wi-Fi, 3G, GPRS and Bluetooth).

## Figures and Tables

**Table 1.** Consumption (in Joules per byte) of symmetric and CP-ABE cryptographic primitives.

**Primitive**	**Energy per Byte**
AES-128/CTR/No Padding Encryption/Decryption	7.62 × 10^−9^
CP-ABE Encryption	1.32 × 10^−6^
CP-ABE Decryption	1.01 × 10^−6^

**Table 2.** Consumption (in Joules) of three popular apps during a time span of 10 min.

**App**	**CPU**	**Comms**	**Display**	**Total**
YouTube	30.11	12.59	508.90	551.59
MX Moto	129.24	5.75	509.54	644.52
Facebook	137.76	27.42	471.42	637.27
